# A Large Sigmoid Pseudo-Pedunculated Lipoma Mimicking an Adenoma: A Report of a Rare Case and Literature Review

**DOI:** 10.7759/cureus.67419

**Published:** 2024-08-21

**Authors:** Raya Abu-Khalaf, Layan Abu-Khalaf, Sandra Abu-Khalaf, Amer Abu-Khalaf

**Affiliations:** 1 School of Medicine, Royal College of Surgeons in Ireland, Dublin, IRL; 2 General Surgery, University Hospital Limerick, Limerick, IRL; 3 Gastroenterology and Hepatology, The Specialty Hospital, Amman, JOR

**Keywords:** lifting sign, iron deficiency anaemia, mimicking adenoma, lipoma, pseudo-pedunculated

## Abstract

Large colonic lipomas exceeding 2.0 cm in size may rarely mimic colorectal adenomas and hence we referred to such lipomas as pseudo-pedunculated lipomas. They can exhibit surface erosions, fissure ulcerations, and mucosal erythema and can pose diagnostic dilemmas during colonoscopy. Our patient presented with iron deficiency anaemia and a positive faecal immunochemical test, a rare but recognised presentation of pseudo-pedunculated lipomas. Available advanced and refined procedures made endoscopic removal of large and even giant, symptomatic, pedunculated lipomas safe and should be the standard of care, while surgical intervention is rarely necessary. Nonetheless, utmost care must be exercised during endoscopic resection due to the high current needed to snare such large lesions to avoid deep thermal injury and perforation. Our case showed that large lipomas can be misinterpreted during endoscopy as adenomas. In experienced hands, endoscopic removal is safe and attainable.

## Introduction

Lipomas are space-occupying lesions that arise in the submucosa and are defined as benign mesenchymal tumours of adipose tissue [1]. Lipomas manifest in different anatomical locations of the body, including skeletal muscles, skin, and the gastrointestinal tract (GIT). Notably, approximately 70% of GIT lipomas occur in the colon, with the right colon being the most frequently affected site, particularly the ascending colon and the caecum [2,3]. They rarely occur in the small intestine, stomach, and oesophagus [4].

Typically solitary, lipomas tend to manifest in individuals aged between 50 and 70 years [5]. However, reports regarding sex predilection in the literature are conflicting [6-8]. Small colonic lipomas are often asymptomatic and are frequently discovered incidentally during colonoscopy. However, lipomas that are more than 2.0 cm in size are rare and can present with symptoms such as abdominal pain, large bowel obstruction due to intussusception, change in bowel habits, and GIT bleeding [6].

On the other hand, colorectal adenomas are far more common than lipomas. After the age of 60, almost every other person undergoing a screening colonoscopy is likely to be diagnosed with colorectal adenomas. They can be small to very large in size and can be found anywhere in the colorectal region. If symptomatic, adenomas can present with occult or rarely overt GIT bleeding and can undergo malignant transformation [9]. Endoscopic ultrasound (EUS) is rarely needed to differentiate between pedunculated lipomas and adenomas. EUS typically shows lipomas as hyperechoic, well-circumscribed lesions with absent or minimal blood flow, and can determine the depth of extension into the muscularis propria where the deeper the depth, the higher the risk of perforation after endoscopic mucosal resection [1].

## Case presentation

A 70-year-old male patient was referred to the gastroenterology clinic for evaluation of mild iron deficiency anaemia (IDA), alternating bowel movements, and abdominal and rectal flatulence. His appetite was normal and he did not report any weight loss. He did not have dyspeptic symptoms and has not reported rectal bleeding, melaena stools, or bleeding from other orifices. Physical examination revealed mild pallor and the abdomen was soft and nontender, with no palpable masses or organs.

Our patient has had type 2 diabetes mellitus (T2DM) and coronary heart disease with multiple coronary stents. Current medications included Glyxambi 10 mg (10 mg empagliflozin and 5 mg linagliptin), bisoprolol 2 mg, baby aspirin 81 mg, and clopidogrel 75 mg daily. The ejection fraction (EF) was 36% and an electrocardiogram (ECG) showed sinus rhythm with old ischemic changes. Ultrasound scan of the abdomen and pelvis was unremarkable.

Laboratory results, as shown in Table 1, revealed a mild degree of IDA and a positive faecal immunochemical test (FIT). His blood sugar, lipids profile, and liver enzyme were slightly deranged.

**Table 1:** Patient's laboratory results.

Test	Result	Reference range
Haemoglobin	12.5	12.0-15.0 g/dL
Mean corpuscular volume	76	82-100 fl
White blood cells	6.5	4.5-11.0 x 10^9^/L
Platelets	250	150-450 x 10^9^/L
International normalized ratio	1.2	0.9-1.2
Serum iron	35	50-150 μg/dL
Total iron-binding capacity	410	250–310 μg/dL
Serum ferritin	15	10-291 ng/ml
Serum B12	680	187-883 pg/ml
Folic acid	8.5	1.8–9.0 ng/mL
Fasting blood sugar	146.0	70.0-99.0 mg/dL
Glycosylated haemoglobin	7.8	4.8-5.7%
Urea	35.00	12.84-43.00 mg/dL
Creatinine	0.9	0.5-1.0 mg/dL
Alanine aminotransferase	49	0-34 u/L
Serum triglyceride	250	<150 mg/dL
Total cholesterol	220	<200 mg/dL
Low-density lipoprotein	130	<100 mg/dL

The patient was evaluated by the anaesthesiologist and he was scheduled for an upper and lower GI endoscopy after stopping clopidogrel. Esophagogastroduodenoscopy (EGD) showed only a small hiatal hernia but erosive changes or gastroduodenal lesions were not detected. Gastric biopsies showed intestinal metaplasia but were negative for dysplasia and neoplasia. Duodenal biopsies did not show histological changes of gluten enteropathy. A total colonoscopy was also performed and bowel preparation was excellent with a total score of 9 on the Boston Bowel Preparation Scale [10].

**Figure 1:**
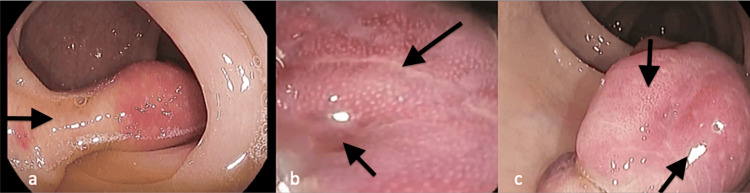
Colonoscopy images showing (a) a large pseudo-pedunculated sigmoid lipoma with marked hyperaemia (b and c) lobular changes, fissuring, ulcerations, and erosions.

A large polypoid lesion was identified in the sigmoid colon located at 35.0 cm from the anal verge. It had a short but rather wide (10.0 mm) stalk and measured 3.5 cm in diameter (Figure 1).

The covering mucosa showed lobular changes, fissures, erosions, and marked erythema (Figure 1). However, the polyp did not have the classical surface blood vessels and pit pattern of conventional adenomas. The cushion sign and the tenting effect were both difficult to elucidate due to surface ulceration and fissuring. The impression was that it was a large lipoma herniating into the sigmoid lumen resembling a pedunculated adenoma (a large sigmoid pseudo-pedunculated lipoma).

**Figure 2:**
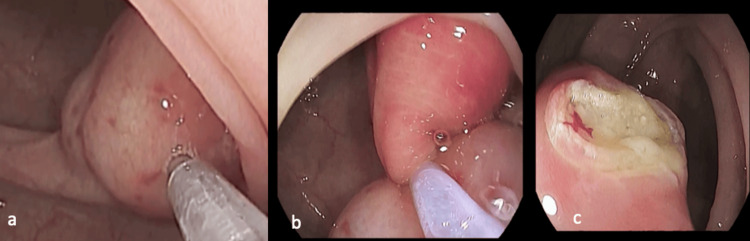
Endoscopic resection of the large pseudo-pedunculated lipoma (a) showing injection of the stalk, (b) the snare in place, and (c) yellowish post-resection margin.

As the lesion was symptomatic, it was decided to attempt endoscopic resection. The stalk was injected with diluted adrenaline and normal saline (NS) and showed adequate lifting (Figure 2). A thin monofilament snare was used (Figure 2) to deliver more current and achieve quicker transection as adipose tissue has lower conduction of electrosurgical current [8].

**Figure 3:**
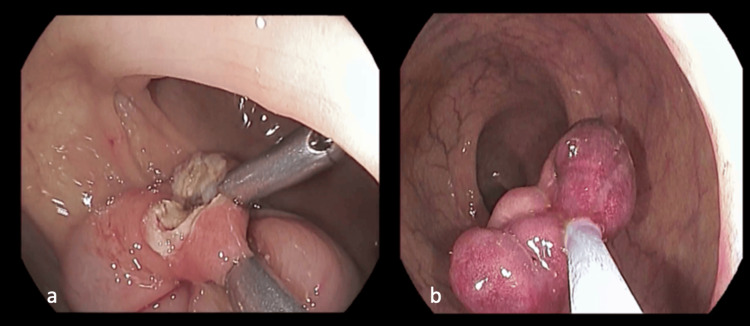
Colonoscopy images showing (a) two endoclips in situ and (b) retrieval of the large, intact pseudo-pedunculated lipoma.

The pseudo-pedunculated lipoma was safely snared (Figure 2) and retrieved (Figure 3). Two endoclips, 16.0 mm each, were applied to the resection site (Figure 3). No immediate or delayed complications were encountered.

**Figure 4:**
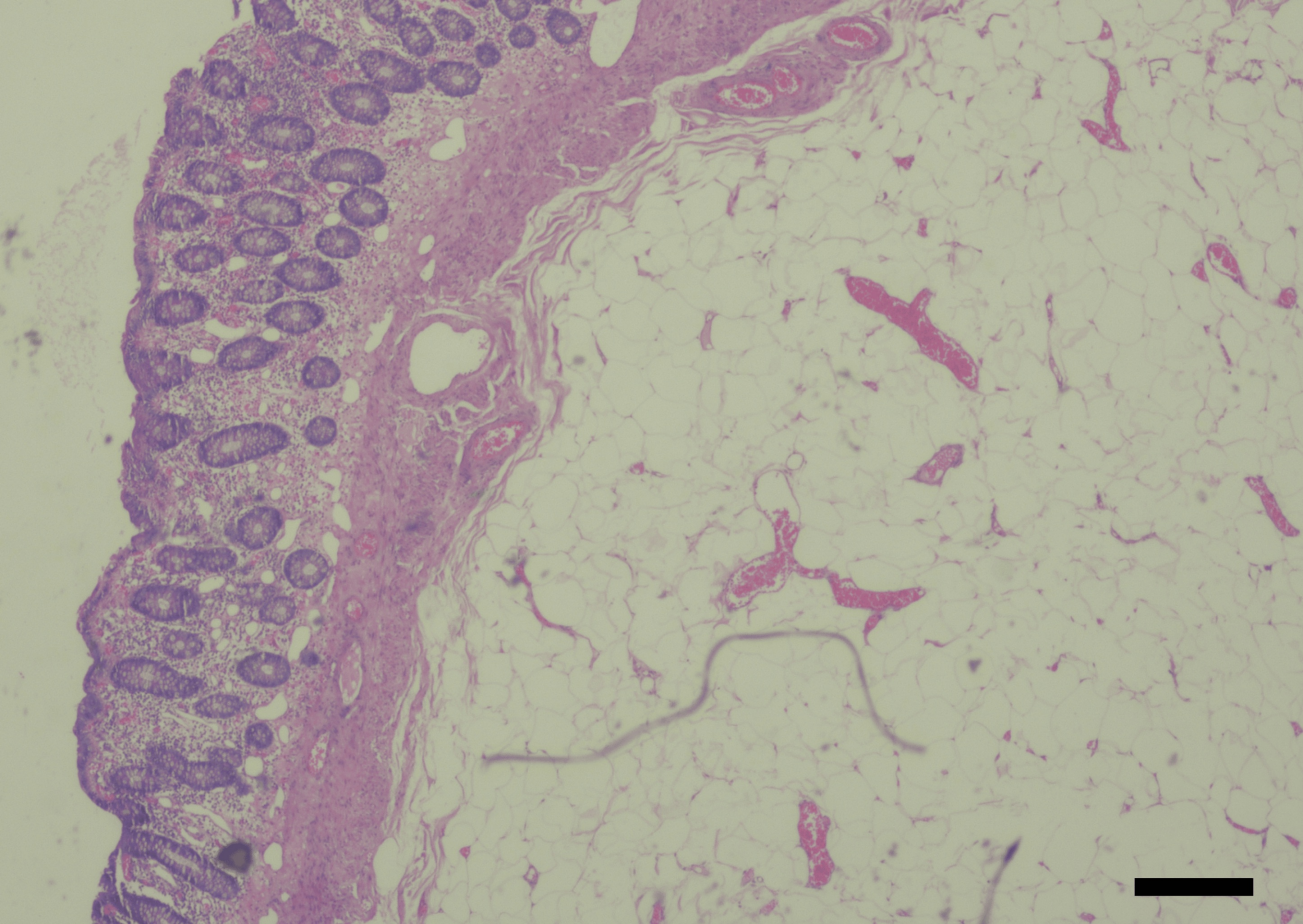
Histopathology sections using H&E stain showing a lobulated mass with congestion and chronic inflammatory cell infiltrate of the overlying mucosa, mature adipose cells, and irregular fibrous septa, typical of a sigmoid lipoma. Hematoxylin & eosin (H&E): 200x; scale bar: 50 μm.

Histopathology sections using hematoxylin and eosin (H&E) stain showed a polypoid lesion covered by colonic mucosa, overlying a lobulated mass composed of mature adipose cells, separated by irregular fibrous septa. The overlying mucosa showed congestion, and a moderate degree of chronic inflammatory cell infiltrate (Figure 4). No cellular atypia and no malignancy were detected. Hence, the diagnosis of a sigmoid colon lipoma was confirmed.

## Discussion

Large colonic lipomas (LCLs) (>2.0 cm) are rare, but small sessile lipomas are more common and are usually discovered incidentally at colonoscopy. LCLs typically have a smooth yellowish surface and a pedunculated thick stalk and are usually positive for tenting, cushion, and naked signs at endoscopy [11]. Our patient’s lipoma showed severe hyperaemia, a lobular ulcerated surface, a nice stalk, and absent tenting and cushion signs, so we referred to it as a pseudo-pedunculated lipoma.

If in doubt, endoscopic ultrasound typically shows the hyperechoic nature of the lipoma and its layer of origin [1]. It is estimated that 90% of colonic lipomas are located in the submucosa while the rest arise from intramucosal or subserosal origin [12]. Colonic lipomas with a maximum diameter of 2.0 cm are labelled as LCL. However, if the size is ≥4.5 cm, they are called giant colonic lipomas (GCLs) [13]. The size and surface characteristics of lipomas will determine their symptomatology although most of these lesions are asymptomatic.

Most lipomas are small in size <2.0 cm, asymptomatic, and are found in the ascending colon and caecum [3]. They are multiple in less than 10% of cases. It seems that both males and females are equally affected [7]. Lipomas that exceed 2.0 cm in size may present with irritable bowel syndrome-like symptoms such as abdominal pain and altered bowel motions [14]. They can also cause IDA and bleeding per rectum due to surface erosions and ulcerations [15]. Hence, LCL must be included in the differential diagnosis of occult GIT bleeding.

Complications of LCL may include intermittent large bowel obstruction, intussusception, and rarely prolapse [14]. Excision of large and even giant submucosal lipomas in experienced hands and selection of the proper endoscopic resection technique is safe and effective. Complications may include perforation, early or delayed bleeding, post-resection serositis, and failure to resect the lesion.

Current endoscopic procedures include hot snare polypectomy meant for lipomas <2.0 cm in size and saline ± adrenaline injection followed by resection using a thin monofilament snare [15]. Further procedures used include endoloop ligation with or without hot snaring, unroofing, endoscopic mucosal resection, complete or piecemeal, and endoscopic submucosal dissection [15-17]. Endoscopic resection must be considered the standard of care except for huge, sessile lipomas arising from the muscular layer.

For safer removal of a lipoma, specific precautions must be taken during endoscopic resection. Adipose tissue lacks water that conducts electricity during endoscopic resection and requires higher current levels and the use of a thin monofilament snare for quick and efficient transection of the stalk. To ensure proper haemostasis and avoid deep wall thermal injury and colonic perforation, we recommend injecting the stalk with saline and diluted adrenaline. This helps to separate the lesion from the muscularis propria. We then employ the “snare traction technique” after targeting the mid-stalk for optimal results [18]. In the literature, there are no specific guidelines on the follow-up of endoscopically resected LCL. However, we suggest a repeat colonoscopy after 12 months to rule out local recurrence.

Endoscopic resection is safe and effective with 0% mortality versus a predicted surgical mortality of 3.3% (p < 0.0001) [19,20]. However, surgical excision is indicated for giant sessile lesions, large bowel obstruction, intussusception, and lesions arising from the muscularis propria and serosa.

## Conclusions

Large pedunculated colonic lipomas can mimic adenomas and pose a diagnostic dilemma during colonoscopy. While colonic lipomas very rarely present with IDA, they should still be included in the differential diagnosis of occult GIT bleeding. Endoscopic removal of LCL should be the standard of care and is proven to be safe and effective. Surgery may be warranted in cases of giant sessile lipomas and for those originating from the muscularis propria or serosa.
